# The impact of the Caring Callers Program on Senior Companion Volunteers and Clients During the COVID-19 Pandemic

**DOI:** 10.1093/geroni/igab046.986

**Published:** 2021-12-17

**Authors:** Kathy Lee, Noelle Fields, Gretchen Feinhals, Melanie Calhoun

**Affiliations:** 1 University of Texas at Arlington, Coppell, Texas, United States; 2 University of Texas at Arlington, Arlington, Texas, United States; 3 The Senior Source, Dallas, Texas, United States

## Abstract

**Purpose of the Study:**

The purpose of this study was to examine the impact of the Caring Callers Program on older adults and volunteers. Our research team piloted this telephone reassurance program during the COVID-19 pandemic. In the Caring Callers Program, socially isolated older adults were paired with older adult volunteers from the Senior Companion Program (20 pairs).

**Methods:**

In the Caring Callers Program, Senior Companion volunteers provided the clients with emotional support through a weekly phone call over the 12 weeks period (May through July 2020). Prior to the intervention implementation, the volunteers received a two-hour group-based training through a teleconference platform. Program outcomes were measured through quantitative and qualitative approaches.

**Results:**

The clients (mean age=73.5) showed significantly increased overall self-rated health at post-test, compared to pre-test and they discussed social and emotional benefits. The clients were very satisfied with the program and indicated that the program met their expectations. Our individual, in-depth interviews with the volunteers (mean age=73.2) also revealed that the volunteers were able to develop their skills that are helpful for their Senior Companion volunteer activities and experience mutual benefits by spending their time more purposefully. Overall, our participants shared that they wanted to continue participating in the Caring Callers Program.

**Discussion:**

We learned the importance of training not only for the volunteers but also for the clients, prior information on their pair, making sure of the volunteer-client fit, and benefits of using telephone particularly in this group of vulnerable older adults.

